# Prevalence and characteristics of Livestock-Associated Methicillin-Resistant *Staphylococcus aureus* (LA-MRSA) isolated from chicken meat in the province of Quebec, Canada

**DOI:** 10.1371/journal.pone.0227183

**Published:** 2020-01-10

**Authors:** Jocelyn Bernier-Lachance, Julie Arsenault, Valentine Usongo, Éric Parent, Josée Labrie, Mario Jacques, François Malouin, Marie Archambault

**Affiliations:** 1 Centre de Recherche en Infectiologie Porcine et Aviaire (CRIPA-FRQNT), Faculté de médecine vétérinaire, Université de Montréal, Saint-Hyacinthe, Québec, Canada; 2 Groupe de recherche sur les maladies infectieuses en production animale (GREMIP), Faculté de médecine vétérinaire, Université de Montréal, Saint-Hyacinthe, Québec, Canada; 3 Groupe de recherche en épidémiologie des zoonoses et santé publique (GREZOSP), Faculté de médecine vétérinaire, Université de Montréal, Saint-Hyacinthe, Québec, Canada; 4 Département de biologie, Faculté des sciences, Centre d'Étude et de Valorisation de la Diversité Microbienne (CEVDM), Université de Sherbrooke, Sherbrooke, Québec, Canada; Instituto de Technologia Quimica e Biologica, PORTUGAL

## Abstract

This study was conducted to estimate the prevalence of Livestock-Associated Methicillin-Resistant *Staphylococcus aureus* (LA-MRSA) in retail chicken meat and broiler chickens from the Province of Quebec, Canada, and to characterize LA-MRSA isolates. A total of 309 chicken drumsticks and thighs were randomly selected in 2013 from 43 retail stores in the Monteregie. In addition, nasal swabs and caeca samples were collected in 2013–2014 from 200 broiler chickens of 38 different flocks. LA-MRSA was not detected in broiler chickens. Fifteen LA-MRSA isolates were recovered from four (1.3%) of the 309 chicken meat samples. Multi-Locus Sequence Typing (MLST) and SCC*mec* typing revealed two profiles (ST398-MRSA-V and ST8-MRSA-IVa), which were distinct using pulse-field gel electrophoresis (PFGE) and microarray (antimicrobial resistance and virulence genes) analyses. In addition to beta-lactam resistance, tetracycline and spectinomycin resistance was detected in all isolates from the 3 positive samples of the ST398 profile. Southern blot hybridization revealed that the resistance genes *aad*(D) and *lnu*(A), encoding resistances to aminoglycosides and lincosamides respectively, were located on plasmid. All isolates were able to produce biofilms, but biofilm production was not correlated with *hld* gene expression. Our results show the presence of two separate lineages of MRSA in retail chicken meat in Quebec, one of which is likely of human origin.

## Introduction

Methicillin-Resistant *Staphylococcus aureus* (MRSA) is a global threat to public health. This bacterium is responsible for a wide range of diseases from superficial skin infections to life-threatening conditions [1]. MRSA are resistant to most beta-lactam antibiotics because the activity of antibiotic-inhibited penicillin binding proteins (PBPs) is replaced by the function of an acquired PBP (PBP2a) with low affinity for these drugs. This low affinity PBP2a is encoded by the *mecA* or *mecC* (a *mecA* homologue) genes located on mobile genetic elements called staphylococcal chromosomal cassettes (SCC*mec*) [2]. Various categories of MRSA have been described based on the source of infection and these include community-associated (CA), healthcare-associated (HA) and livestock-associated (LA) MRSA [3]. About 15 years ago, a LA-MRSA of multilocus sequence type (ST) 398 has emerged in food production animals [4–6]. Pigs are considered as reservoirs of this MRSA lineage, with high prevalence documented by several reports [7–10]. Colonization by ST398 has also been reported in poultry [11–13] and cattle [14]. Workers in close contact with pigs are at significant risk for colonization by ST398 [5, 8, 15–17], supporting its zoonotic potential and raising questions regarding food safety. Also, whole genome analysis of LA-MRSA ST398 has indicated several mobile genetic elements that confer antibiotic resistance [18] as well as the presence of multiple integrative conjugative elements combined with the absence of a type I restriction and modification system which could enhance horizontal gene transfer [19].

In various European countries, prevalence of LA-MRSA ranging from 0 to 16% in broiler chickens [11, 20–22] and from 0 to 37% in chicken retail meat products [20, 22–24] have been reported. Most of these LA-MRSA isolates were identified as ST398. In Hong-Kong, MRSA was isolated from 6.8% of 455 chicken meat samples purchased at retail outlets; most isolates belonged to ST9 while only one isolate was identified as ST398 [25]. In a Canadian study, MRSA was identified in 3 (1.2%) of 250 chicken meat samples tested [26]. They were classified as Canadian epidemic MRSA-2 (CMRSA-2), also known as USA100, recognized as the most common cause of HA-MRSA infection in Canada [27]. In Detroit, MRSA was detected in 3 (4%) of 76 retail chicken samples, all identified as ST8 [28].

One major characteristic of *S*. *aureus* is its ability to form biofilms [29, 30], which could favor environmental persistence and thus dissemination in farm animals, or increase survival in meat products. Microbial surface components recognizing adhesive matrix molecules (MSCRAMMs) participates in biofilm attachment, whereas polysaccharide adhesion molecule encoded by the *icaABCD* operon [31] are involved in biofilm maturation. The activation of the Agr regulatory system plays a role in biofilm dispersal, which can be evaluated by measuring *hld*-RNA III expression levels [32]. Genes involved in biofilm formation were recently identified in MRSA isolates of poultry origin though no studies were carried out to evaluate the capacity of these isolates to form biofilms [33].

The current study was conducted to estimate the prevalence of LA-MRSA in retail chicken meat and chicken broilers in Quebec and to characterize these isolates with particular reference to their genotypic, virulence, antimicrobial resistance patterns as well their ability to form biofilms.

## Materials and methods

### Sample collection

A two-stage sampling method was used for selecting samples from retail stores and slaughterhouses. Every week between June 2013 to November 2013, 43 retail stores located in the Monteregie region in the Province of Quebec were randomly selected (with replacement) from all retail stores listed in a business phone directory (Yellowpages^TM^) and Google Maps websites under the keywords *alimentation* (food), *marché* (market), *supermarché* (supermarket), *boucherie* (butcher's shop), *épicerie* (grocery). Selected stores were visited at least three times and many of them were visited 5 to 6 times to reach the selected number of samples. The sample size for chicken meat was determined to be at least 280 samples based on an expected prevalence of 3%, an accepted error of 2% and a 95% level of confidence. At the time of the visit, we collected the first package of fresh chicken drumsticks and/or fresh thighs with skin, available at the front line of the shelf. The retail chicken samples were transported on ice to the laboratory where they were processed immediately.

From March to December 2014, one (out of two) large slaughterhouse was visited once every two weeks. The slaughterhouse workers selected one or two Quebec broiler chicken lots and collected five chickens in different cages for each lot. The full street addresses of the chicken barns of origin were collected. The sample size for broiler chickens was set to 200 chickens based on an expected prevalence of 2%, an accepted error of 2% and a 95% level of confidence. These broilers chickens were transported alive in ventilated cages to the Faculty of Veterinary Medicine, Université de Montréal. They were anesthetized with an intramuscular injection of a combination of ketamine (25 mg/kg) and xylazine (2,5 mg/kg), then euthanized using cervical dislocation and sampled. For each chicken, swabs of the choana and of the caecum were carried out, totalling 400 swabs. The protocol was approved (14-Rech-1690) by the Université de Montréal ethics and animal use committee.

### Isolation of bacteria

MRSA isolation from retail chicken samples was conducted as previously described [24] with some modifications, mainly associated with the usage of different selective MRSA plates. Briefly, 25 g of chicken meat with skin were homogenized for two min with a stomacher in 225 mL of Mueller-Hinton supplemented with 6.5% w/v of NaCl and incubated for 16 to 20 h at 37°C. A volume of 1 mL of this culture was added to 9 mL of Tryptic Soy Broth (Oxoid, Wessel, Germany) supplemented with 3,5 μg/mL of cefoxitin and 75 μg/mL of aztreonam and incubated 16 to 20 h at 37°C with agitation for selective enrichment of MRSA. A volume of 10 μL of this culture was inoculated on MRSA select^TM^ agar (Bio-Rad, Mississauga, Ontario, Canada) and incubated for 24 h. Three to four colonies were kept for PCR confirmation. A positive in-house control laboratory strain 100N (LA-MRSA of porcine origin ST398-V) was used to spike meat samples and revealed 1 CFU/25 g as the detection limit. Chicken swabs were inoculated in 10 mL of Mueller-Hinton supplemented with 6.5% w/v of NaCl. Colonies were confirmed as MRSA by multiplex polymerase chain reaction (PCR) detection of *nuc* and *mecA* as previously described with slight modifications [34, 35]. Master mixes were at a final concentration of 1X PCR buffer, 0.16 mM dNTPs, 2 mM of MgCl_2_, 0.12 μM of each primer, 1 U of Taq DNA polymerase (GE Healthcare) and 3 μL of DNA template. An isolate was considered as a MRSA if it carried both *nuc* and *mecA*. In addition, PCR for *mecC*, a *mecA* homologue, was also carried out as previously described by Cuny *et al* [36] with the following condition: initial denaturation step of 2 min at 95°C followed by 30 cycles of 94°C for 30 s, 52°C for 30 s, and 72°C for 30 s, and a final extension at 72°C for 4 min. Primer sequences used in this study can be found in S1 Table.

### Molecular typing of isolates

MLST was performed according to the protocol of Enright *et al* [37]. SCC*mec* typing was done according to the method described by Zhang *et al* [38] with some modifications. Each SCC*mec* type was tested with uniplex PCR instead of multiplex PCR. SCC*mec* types I, II, III, IVd were tested with 25 μL of master mix at a final concentration of 1X PCR buffer, 0.3 mM of each dNTPs, 0.6 mM of MgCl_2_ and KCl, 0.8 μM of each primer, 1.5 U of Taq DNA polymerase (GE Healthcare) and 5 μL of DNA template. SCC*mec* types IVa, IVb, IVc, V were tested with 25 μL of master mix at a final concentration of 1X PCR buffer, 0.3 mM of each dNTPs, 0.6 mM of MgCl_2_ and KCl, 0.28 μM of each primers, 2.0 U of Taq DNA polymerase and 5 μL of DNA template.

### PFGE typing of isolates

PFGE of total DNA was performed using of *SmaI* and *Cfr9I* restriction enzymes (Fermentas Life Sciences, Burlington, ON, Canada) using the protocol of Mulvey *et al* [39]. Stain relatedness was analyzed with *Cfr91* because of the non-typeability of ST398 strains with *SmaI* due to a restriction/methylation system. For the *Cfr9I* restriction enzymes, the incubation time was 4 h at 37°C instead of 2 h at 37°C.

### Antimicrobial susceptibility testing

The minimal inhibitory concentration (MIC) of antimicrobial agents was determined using the broth microdilution method (plates GPN3F and AVIAN1F) with the ARIS automatic system of SensititreTM (TrekTM Diagnostic System Ltd, Cleveland, Ohio, USA). In addition, the Clinical and Laboratory Standard Institute (CLSI) broth microdilution method [40, 41] was used with plates prepared in-house for fosfomycin, tobramycin, spectinomycin, sulfathiazole, ceftiofur and oxytetracycline. Resistance was determined by the CLSI interpretation breakpoints [40, 41]. Specific breakpoints for staphylococci were used when available. For neomycin and tobramycin antimicrobials, the gentamicin staphylococci breakpoint (≥ 16 μg/mL) was used. Otherwise breakpoints for other aerobic microorganisms were applied: breakpoints of *Pasteurella multocida* were used for florfenicol (≥ 8 μg/mL), spectinomycin (≥ 128 μg/mL) and tylosin (≥ 32 μg/mL as indicated for tilmicosin, another 16-membered ring macrolide). Fosfomycin non-susceptibility breakpoint (> 32 μg/mL) of the European Committee on Antimicrobial Susceptibility Testing guidelines (EUCAST) was selected [42]. The *S*. *aureus* ATCC 29213 was used as the control strain for antimicrobial susceptibility testing.

### DNA microarrays

Isolates were characterized by using the StaphyType array based on the Array-Tube platform (Alere technologies, Jena, Germany) [43–45]. This array used a set of probes that were able to detect *S*. *aureus* specific genes, accessory gene regulator (*agr*) alleles, genes coding for virulence factors (toxins, enterotoxins, putative toxins, hemolysins, proteases and biofilm formation molecules), MSCRAMMs, capsule type-specific genes, antimicrobial resistance genes as well as probes for the discrimination of MLST and *spa* types.

### Plasmid DNA extraction

Plasmid DNA was extracted by alkaline lysis using the plasmid midi-Kit (Qiagen) with minor modifications. Cells were grown overnight at 37°C in tryptic soya broth (TSB) and diluted to an *A*_600nm_ of 0.01 in prewarmed medium. They were grown to an *A*_600nm_ of 3 at which time an aliquot of cells was recovered for plasmid extraction. Cells were pelleted by centrifugation and resuspended in buffer P1 supplemented with 100 mL lysostaphin 0.5 mg/mL and the cell suspension was incubated for 1 h at 37°C. Subsequent isolation steps were then followed according to the manufacturers recommendations.

### Southern blot hybridization analysis

Plasmid DNA was digested with 50 U of Hind III (New England Biolabs) for 1 h at 37°C and separated by electrophoresis for 3 h at 45 V in 0.8% agarose. After migration, the digested plasmids were transferred to positively charged nylon membranes (Roche Diagnostics, Mannheim, Germany) using a vacuum blotter model 785 (Bio-Rad). The membranes were probed with digoxigenin labelled PCR products for the genes *aad*(D), *lnu*(A), *spc*, *tet*(K) and *tet*(M) [46] using the PCR DIG probe synthesis kit (Roche Diagnostics) (S1 Table). Pre-hybridizations and hybridizations were carried out at 65°C for 30 min and 18 h, respectively, in hybridization buffer with subsequent washes, as recommended by the manufacturer. To detect the presence of digoxigenin-labelled probes, the colorimetric method (NBT/BCIP substrate solution, Roche Applied Science) was used. PCR products were used as hybridization control and control DNA DIG-labelled as detection control.

### Biofilm formation assay

Biofilm formation was assayed in 96-well microtiter plates as described by Tremblay *et al* [47]. Briefly, isolates were streaked on Brain Heart Infusion (BHI) agar and incubated for 16 h at 35°C. Colonies were harvested and resuspended in BHI broth supplemented with glucose (0.25% w/v) to a 0.5 McFarland standard. A volume of 200 μL of this inoculum was transferred into 3 wells followed by incubation of the microtiter plate at 35°C for 24 h. After 24 h incubation, the growth medium was aspirated and the planktonic cells were removed by washing the wells thrice with phosphate-buffered saline (PBS). The wells were air-dried and then stained with 0.1% (w/v) safranin for 10 min. The wells were washed once with water and air dried at 35°C for 15 min. Safranine was then released with 200 μL of destaining solution (50% (v/v) ethanol, 50% (v/v) glacial acetic acid) for 10 min and the amount of stain released was quantified by measuring the absorbance at 490 nm with a microplate reader (Powerwave, Bio-Tek, Instruments). *Staphylococcus epidermidis* ATCC35984 (a high biofilm producer) and ATCC12228 (a weak biofilm producer) were used as controls. Three in-house control laboratory strains were also tested: USA300 (CA-MRSA ST8-IVa) of human origin, MRSA COL (HA-MRSA) and 100N (LA-MRSA of porcine origin ST398-V). Isolates were categorized according to the biofilm production (none, weak, moderate or strong) based on the absorbance of their 3 replicates relative to the negative control of the plate using a previously elaborated scale [48].

### Quantitative Expression of *hld* (two step qRT-PCR)

Quantitative expression evaluation of *hld* was performed as previously described [49] with minor modifications and used as a surrogate for evaluating Agr activity. Primer sequences can be found in S1 Table. Briefly, overnight cultures of MRSA isolates were used to inoculate BHI broth to an *A*_600nm_ of 0.1. Planktonic cultures were then grown at 35°C until they reach *A*_600nm_ of 0.4. At this absorbance, 2.5 mL of the culture was harvested and treated with RNA protect (Qiagen, Mississauga, ON) followed by bacterial lysis with lysostaphin (200 μg/mL) for 1 h at 35°C. RNA was then extracted from the lysed bacterial cells using the RNeasy extraction kit (Qiagen). One microgram of total RNA was reverse transcribed with 0.5 m*M* of dNTP, 50 ng of random hexamers, and 200 U of Life Technologies Superscript II reverse transcriptase [50]. RNA was hydrolyzed and the cDNAs were purified with QIAquick PCR purification kit (Qiagen). The Stratagene MX3000P Real-Time PCR (Stratagene, La Jolla, USA) was used to amplify one μL of cDNA with a published master mixed [50]. The following conditions were used for the Real-Time PCR reaction: denaturation 10 min at 95°C, followed by 35 cycles of 1 min at 60°C, 1 min at 72°C and finally a dissociation ramp from 55°C to 95°C. The level of expression of the *hld* gene was calculated with the expression fold (2^-ΔCt^) where ΔCt is the variation between Ct of each isolate and Ct of reference strain. The ΔCt of an isolate/reference strain corresponded to the difference between the Ct of the *hld* gene and the Ct of the *gyrB* gene of the same isolate. The *gyrB* gene was found to be constitutively expressed during growth up to early stationary phase and was thus used as a calibrator [51]. The strain 100N (porcine ST398 MRSA) was used as a reference standard for *hld* expression. It is a weak biofilm producer with high expression of *hld* gene.

### Statistical analysis

Prevalence with 95% confidence limits of MRSA-positive chicken meat and poultry samples were estimated. Estimates were adjusted for potential flock (chickens) or retail stores (meat) clustering. For chickens, estimates were also adjusted for sampling probabilities within flocks based on the flock size. In the absence of positive samples, 95% exact confidence limits (Clopper-Pearson) were estimated. Each sample was geolocated using the farm or the retail store address with GeoPinpoint suite version 6.4 (DMTI Spatial). The geographical distribution of samples according to their MRSA status was mapped using administrative boundary vector files from Statistics Canada (2011 census), performed in ArcGIS version 10.5.1. (ESRI).

For each positive meat or chicken sample, the biofilm production of biological replicates (averaged over the 3 technical replicates) was compared between isolates using the exact Kruskal-Wallis test [52]. If no difference was observed, one isolate was randomly selected and kept as a representative member of the sample. Difference in biofilm production (averaged across the 3 technical replicates) of biological replicates was compared between samples (based on one randomly selected isolate per sample and including the USA300, MRSA COL and 100N reference strains) using the exact Kruskal-Wallis test. If the test was significant (p<0.05), post-hoc Nemenyi pairwise comparison tests were performed (alpha = 0.05). Difference in *hld* gene expression between these samples and reference strains was also evaluated using the same method. Correlation between biofilm production and *hld* gene expression of random isolates selected was evaluated with a Spearman correlation test. All statistical analyses were done in SAS software, version 9.4 (Cary, NC, USA).

## Results

### MRSA prevalence in chicken meat and broiler chickens

**Fig 1:**
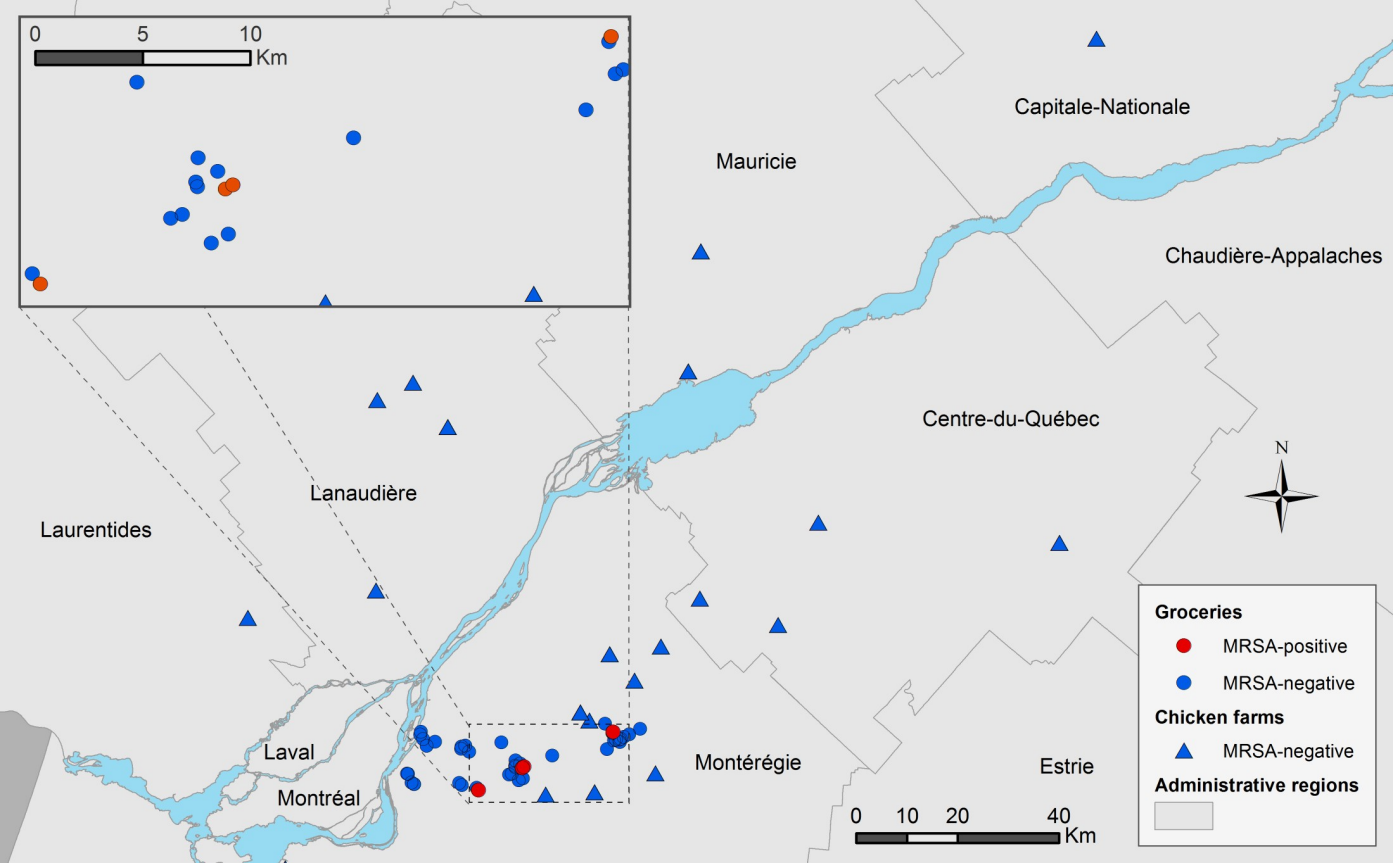
Geographical distribution of retail markets and farms sampled according to the presence of MRSA-positive samples, province of Quebec, Canada, 2013–2014.

A total of 309 retail chicken samples (274 drumsticks, 33 thighs and 2 drumsticks and thighs) were collected from 43 retail stores. MRSA was found in 4 samples (three drumsticks and one thigh) out of the 309 retail chicken meat samples for an estimated prevalence of 1.3% (CI 95%: 0.35% - 3.3%). All 4 positive samples were found during the month of July 2013 and were collected from 4 different retail stores. One to five isolates were kept per positive sample for a total of 15 MRSA isolates (Table 1). MRSA was not detected within the 400 nasal and caecal swabs taken from 200 chicken broilers sampled at two slaughterhouses representing 38 farms, giving an estimated prevalence of positive chickens of 0% (CI 95%: 0.00–1.83%) for each type of sample. The geographical distribution of retail markets and farms of origin according to the MRSA status of samples is illustrated in Fig 1.

**Table 1:** Retail chicken meat MRSA isolates (*n = 15)* profiles based on antimicrobial resistance, pulsotypes, antimicrobial resistance genotypes, virulence genotypes and biofilm formation.

Sample identification			E154	E272					E372				E452				
Isolate identification			a	a	b	c	d	e	a	b	c	d	a	b	c	d	e
MLST-SCC*mec*																	
	ST398-V		●	●	●	●	●	●	●	●	●	●					
	ST8-IVa												●	●	●	●	●
PFGE profile			A	A	A	A	A	A	A	A	A	A	B	B	B	B	B
Phenotypic resistance by class and agent (Red: Resistant; Yellow: Intermediate; Green: Susceptible)																	
	Beta-lactams																
		Amoxicillin															
		Ampicillin															
		Ceftriaxone															
		Oxacillin															
		Penicillin															
		Ceftiofur															
	Tetracyclines																
		Oxytetracycline															
		Tetracycline															
	Aminocyclitol																
		Spectinomycin															
	Aminoglycosides																
		Amikacin															
		Gentamicin															
		Kanamycin															
		Neomycin															
		Streptomycin															
		Tobramycin															
	Lincosamides																
		Clindamycin															
	Lipopeptide																
		Daptomycin															
	Macrolides																
		Erythromycin															
	Tylosin tartrate																
	Fluoroquinolones																
		Ciprofloxacin															
		Enrofloxacin															
		Gatifloxacin															
		Levofloxacin															
	Phenicols																
		Florfenicol															
	Linezolid																
	Streptogramins																
		Quinupristin/dalfopristin															
	Rifamycins																
		Rifampin															
	Novobiocin																
	Potentiated sulfas																
		Sulphadimethoxine															
		Sulphathiazole															
		Trimethoprim/sulfamethoxazole^a^															
	Glycopeptides																
		Vancomycin															
	Fosfomycin																
AMR genes by expected resistance^b^ (Red: Present; Green: Absent)																	
	Beta-lactams																
		*mecA*															
		*blaZ*															
	Fosfomycin, bleomycin																
		*fos*B															
	Macrolides, lincosa-mides, streptogramins																
		*rm*(A)															
	Tetracyclines																
		*tet*(M)															
		*tet*(K)															
	Lincosamides, pleuro-mutilins, streptogramin																
		*lin*(A)															
	Quaternary ammonium																
		*qacC*															
	Spectinomycin																
		*spc*															
	Aminoglycosides (tobramycin, neomycin)																
		*aad*(D)															
Virulence gene by virulence factors^b^ (Red: Present; Green: Absent)																	
	Leukocidin/ hemolysins/aureolysins																
		*lukS*, *lukF*, *hlgA*, *lukX*, *lukY*, *hla*, *hlb*, *aur*															
		*lukf-PV*, *luks-PV*, *lukD*, *lukE*															
	Enterotoxins																
		*entK*, *entQ*															
	Immunoevasion																
		*isdA*															
		*sak*, *chp*, *scn*															
	Arginine catabolic mobile element locus																
		*arcA*, *arcB*, *arcC*, *arcD*															
	Regulatory gene																
		*agrI*, *agrB-I*, *agrC-I*, *agrD-I*, *hld*															
	Adherence/biofilm																
		*icaA*, *icaC*, *icaD*															
	Superantigen-like proteins																
		*setC*, *setB1*															
		*setB2*, *setB3*															
	Microbial surface components recognizing adhesive matrix molecules/Adhesion																
		*bbp*, *clfA*, *clfB*, *ebpS*, *fnbA*, *fnbB*, *map*, *sdrC*, *sdrD*, *vwb*															
		*sasG*, *fib*															
		*cna*															
Biofilm formation (Red: High; Yellow: Moderate; Green: Weak)																	

^a^ The MIC values of trimethoprim/sulfametoxazole (0.5/9.5) are given as trimethoprim MIC values.

^b^ StaphyType array (Alere technologies, Germany); Microarray identifies an overall hybridization pattern that is in accordance to a CC or ST and carries probes for the discrimination of selected SCC*mec* types.

### Molecular typing of retail chicken meat MRSA isolates

**Fig 2:**
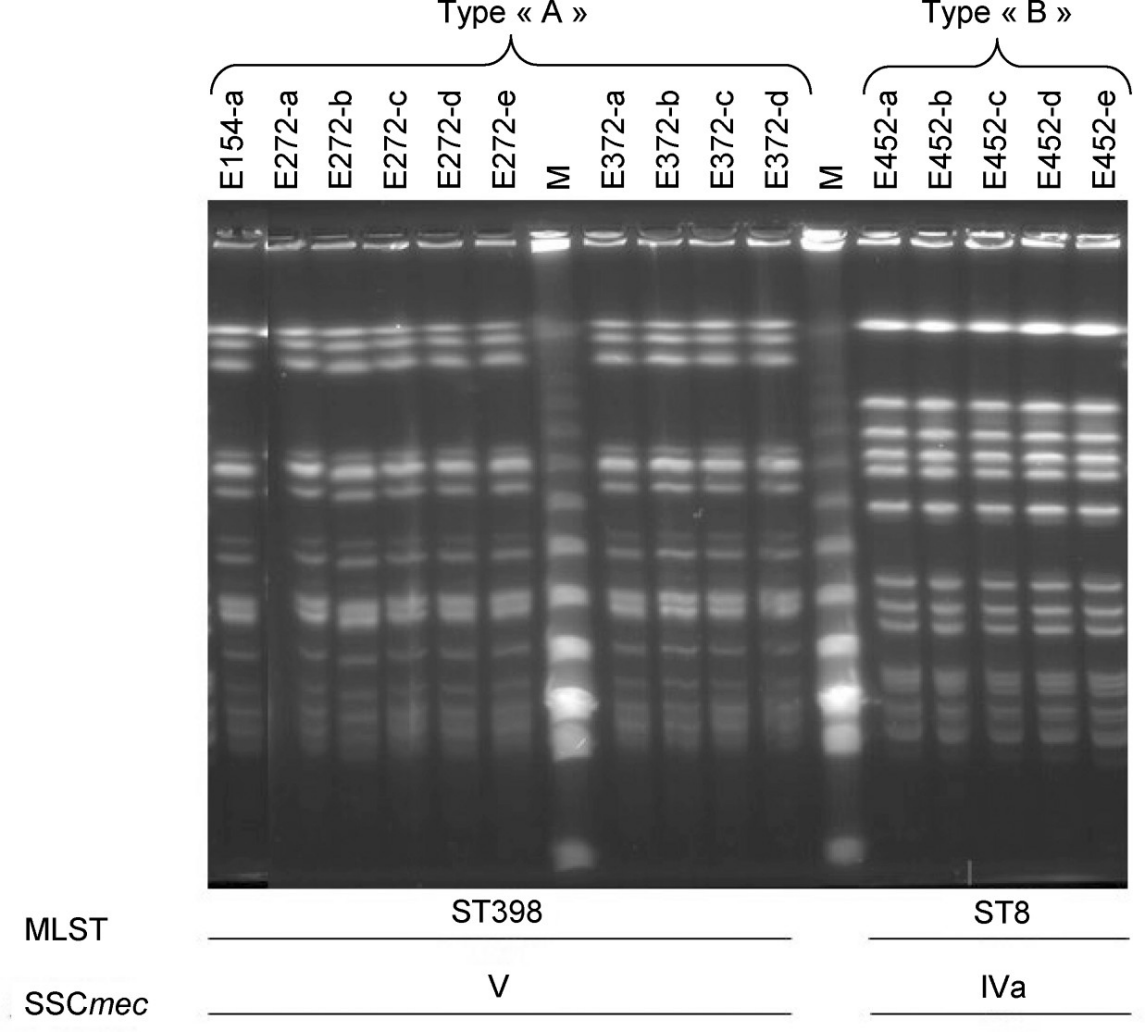
Pulse-field gel electrophoresis following *Cfr9I* digestion. M: lambda DNA marker, Type “A”: Pulse-field gel type A, Type “B”: Pulse-field gel type B.

Multi-Locus Sequence Typing (MLST) revealed the presence of 2 different sequence types among the isolates; ST398 (*n* = 10 isolates from three positive meat samples) and ST8 (*n* = 5 isolates from one positive meat sample) (Table 1). SCC*mec* typing identified two types of cassettes; V for the ST398 isolates and IVa for the ST8 isolates. All the ST398 isolates were non-typeable by PFGE using *SmaI* but were all typeable using *Cfr9I*. Isolates were divided into 2 distinct patterns: type A (ST398 isolates) and type B (ST8 isolates). Isolates in each pulse-field type displayed indistinguishable patterns (Fig 2).

### Antimicrobial resistance phenotypes

All isolates were susceptible to amikacin, gentamicin, kanamycin, neomycin, streptomycin, clindamycin, daptomycin, erythromycin, tylosin tartrate, ciprofloxacin, enrofloxacin, gatifloxacin, levofloxacin, linezolid, quinupristin/dalfopristin, rifampicin, novobiocin, sulfadimethoxime, sulfathiazole, trimethoprim/sulfamethoxazole, vancomycin and fosfomycin (Table 1). All isolates were intermediate or resistant to beta-lactams, spectinomycin, and florfenicol. Resistance to tetracycline and tobramycin was only observed in ST398 isolates. Considering the latter results, multidrug resistance, defined as intermediate or complete resistance to 3 or more classes of antimicrobials, was present in all isolates.

### Microarray analysis

According to the microarray analysis, in addition to the *mecA* gene, ST398 isolates harboured several antibiotic resistance genes responsible for macrolides, lincosamides, tetracyclines, aminoglycosides, and aminocyclitols resistances (Table 1). The presence of these genes correlated well with the phenotypic antimicrobial resistances observed with the exception of florfenicol resistance which could not be explained by the florfenicol/chloramphenicol resistance genes *fexA* and *cfr*. All isolates (*n* = 15) were positive for genes (*hlgA*, *lukF/hlgB*, *lukS/hlgC)* encoding components of leucocidin toxin *γ-*hemolysin as well as other putative leucocidin toxin genes *(lukX*, *lukY*) previously observed in CA-MRSA [53]. Genes *hla* and *hld* coding for α and δ hemolysins, superantigen-like genes (*setC* and *setB2*), genes indicative of capsule type 5, *sarA* as well *icaACD* genes that encode proteins involved in biofilm formation were carried by all isolates. In addition, all isolates carried a similar set of genes for MSCRAMMs including *clfA* and *clfB* (encoding clumping factor A and B) *fnbA* and *fnbB* (encoding fibronectin proteins), *ebpS* (encoding elastin binding protein) among others. All isolates were negative for exfoliative toxin genes, the toxic shock syndrome toxin 1 *tst* alleles, and the enterotoxin gene cluster *egc*. Contrary to ST398 isolates, all ST8 isolates were negative for *cna* (encoding collagen-binding protein) gene, but positive for 1) *sasG* (encoding a cell wall anchored surface protein); 2) *entK* and *entQ* encoding enterotoxin K and Q; 3) *arcA*, *arcB*, *arcC* and *arcD* of the arginine catabolic mobile element locus; 4) *sak*, *chp* and *scn* of the immuno-evasion cluster encoding immune modulation proteins; 5) Panton-Valentine leukotoxin genes *lukf-PV* and *luks-PV* [53, 54], and 6) superantigen-like genes *setB2* and *setB3*. That being said, given that the ST8 isolates were PVL-positive and positive to arginine catabolic mobile element (as highlighted in Table 1), these are likely USA300 strains.

### Plasmid and Southern blot hybridization analysis

**Fig 3:**
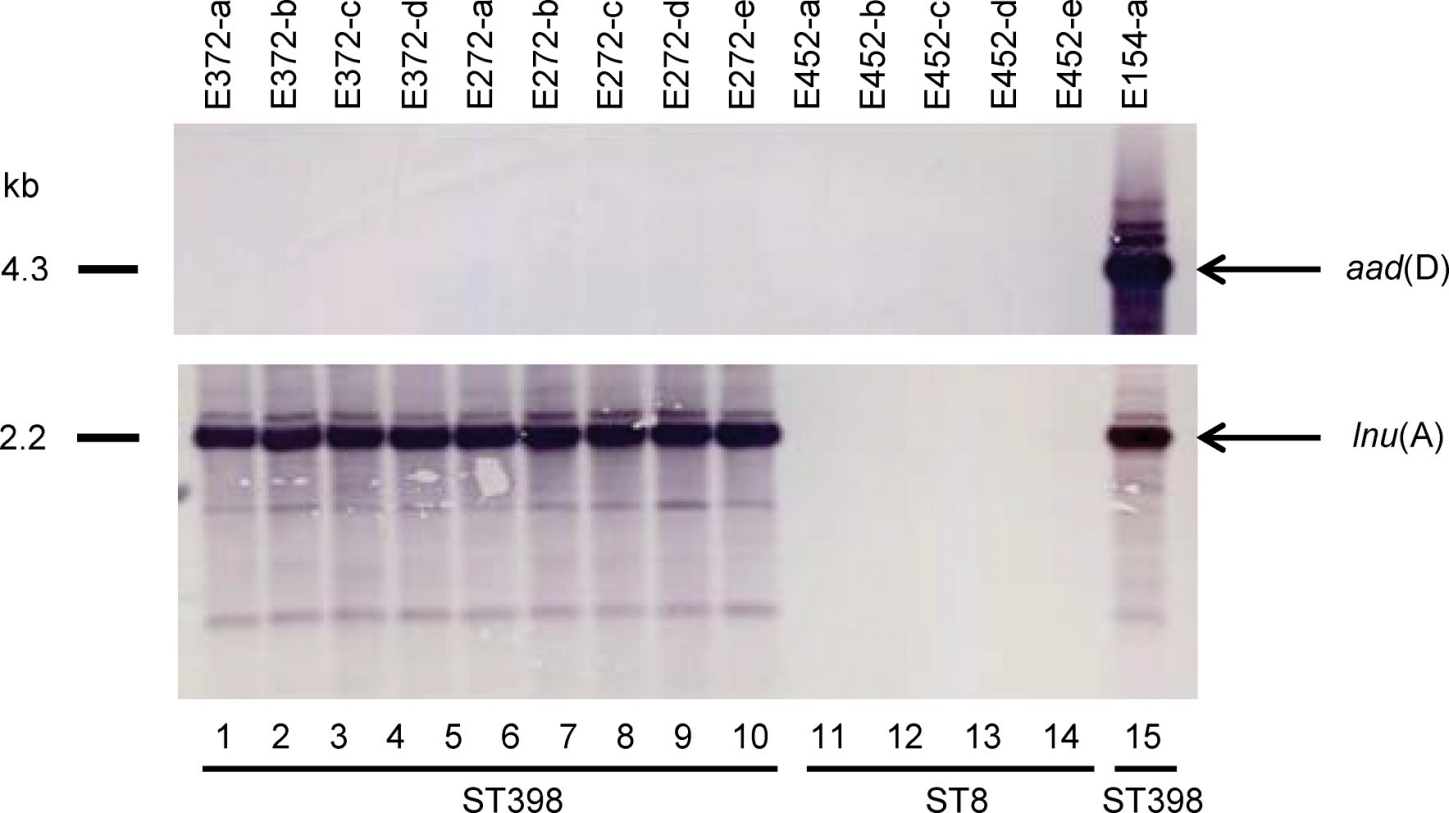
Plasmid location of *aad*(D) and *lnu*(A). Southernblot hybridization of plasmid DNA with DIG labelled probes for *aad*(D) top panel and *lnu*(A) bottom panel. Kb represents kilobase pairs.

The plasmid and southern blot hybridization analysis was carried out to address the presence of mobile genetic elements associated with antimicrobial resistance and possible co-localization of genes. Plasmid extraction and analysis revealed that the resistance gene *lnu*(A) was located on three plasmid bands of approximately 2.2 kb in all ST398 isolates whereas the *aad*(D) gene was located on four plasmids of approximately 4.3 kb in one ST398 isolate (Fig 3). The number of bands may be explained by incomplete digestion of the circular plasmids. The *spc*, *tet*(K), and *tet*(M) genes were not located on plasmid.

#### Biofilm formation

**Fig 4:**
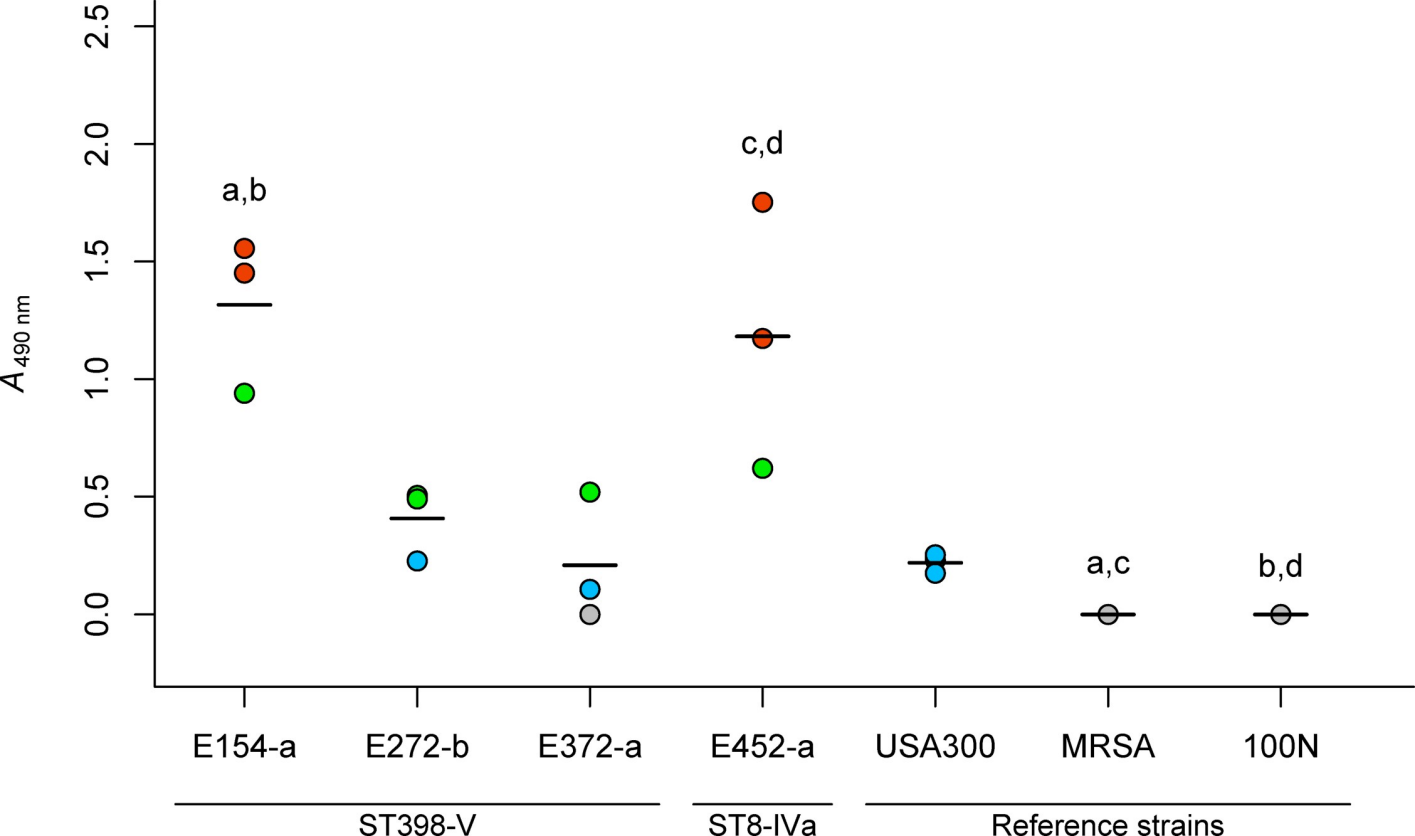
Biofilm production in retail chicken meat MRSA isolates and reference strains. Dots represent mean *A*_490 nm_ of the three technical replicates for each biological replicate; line is the mean *A*_490 nm_ of the three biological replicates. ^a^ Statistical difference between E154-a and MRSA COL (*p* < 0.05). ^b^ Statistical differences between E154-a and 100N (*p* < 0.05). ^c^ Statistical difference between E452-a and MRSA COL (*p* < 0.05). ^d^ Statistical difference between E452-a and 100N (*p* < 0.05). Red dot, strong biofilm production; green dot, moderate biofilm production; blue dot, weak biofilm production; gray dot, no biofilm production.

Biofilms have been associated with adherence to biotic and abiotic surfaces, tolerance to antimicrobials and persistence into the environment [55]. Thus, this analysis was carried out to understand if the recovered MRSA of meat origin were biofilm producers. All isolates were able to form biofilm using the microtiter plate assay (Table 1 and S1 Fig). Biofilm formation was classified as absent in all the reference strains tested, except for *S*. *epidermidis* ATCC 35984, a high biofilm producer (S1 Fig). No significant statistical difference in biofilm formation (p > 0.05) was found between isolates from the same positive sample. Thus, one per positive samples (n = 4) was randomly selected for further statistical analyses. Biofilm formation was classified as high in most replicates of isolates E154-a and E452-a and as weak or moderate in most replicates of isolates E272-a and E372-a (Fig 4). Overall, biofilm formation was significantly different between isolates, including the 3 reference strains (p = 0.01). However, according to post-hoc test, no significant statistical difference between biofilm production of MRSA ST398 and ST8 isolates was observed.

### *hld* gene expression

The expression of the *hld* gene was measured by quantitative real time PCR using the same isolates (E154-a, E272-b, E372-b, E452-b) that were used for the biofilm assay (S2 Fig). No significant statistical difference (p = 0.06) in expression of the *hld* gene compared to the expression of the reference strain 100N was seen between the isolates, either when including or excluding the reference strain (p = 0.78). No correlation was observed between biofilm production and *hld* gene expression (Spearman correlation coefficient = 0.2, p = 0.80).

## Discussion

This study documented the prevalence of LA-MRSA in broilers and retail chicken meat in the province of Quebec and characterized the isolates recovered. Surprisingly, in contrast to studies from Belgium [11, 13], Netherlands [21] and Poland [22], we did not find LA-MRSA in broilers. The anatomical sites sampled were selected to optimize detection sensitivity, based on a Belgian study reporting that MRSA was isolated most frequently from nares and cloacae [56]. However, we cannot ruled out that some positive samples were missed by the birds being positive in other sites or because the double enrichment procedure we used was too selective for MRSA detection in a low-prevalence population, indicating that we may have selected against heterogenous MRSA strain with low cefoxitin MICs [56, 57].

The LA-MRSA prevalence of 1.3% in retail chicken meat in this study is similar to findings in another region of Canada [26], the USA [28, 58, 59] and Poland [22], but lower than prevalence obtained in Netherlands [23] and Germany [24]. The underlying causes of these regional variations in the prevalence of MRSA in broilers and chicken meat has not been explored to our knowledge. At the farm level, differences in flock size, antimicrobial drug use, animal movement or type of housing might be involved as reported for pig farms [60].

Our isolates belonged to two distinct sequence types: ST398 and ST8. Studies investigating MRSA in retail meat products documented ST398 as the predominant MRSA [23, 61]. ST8 has also been found in retail meat products including chicken as well as pork, beef and turkey [28, 59, 62, 63]. Isolates from 3 samples belonged to SSC*mec* type V, which has been found in retail meat samples worldwide [23–25, 58, 64–66]. Isolates from the remaining sample belonged to SSC*mec* type Iva. This type has also been found in retail meat samples in the United States [28, 59, 63]. All isolates within each ST demonstrated indistinguishable pulse-field pattern type reflecting clonality, further confirming the MLST results.

Microarray data reveal that none of the MRSA isolates in this study harbour genes encoding exfoliative toxin and toxic shock syndrome toxin 1. These toxins were not identified in MRSA isolates of retail chicken meat in Germany [24] in accordance with our findings. Moreover, these genes have been rarely detected in LA-MRSA ST398 isolates [43–45, 67]. Whereas all the ST8 isolates were positive in the microarray for the *lukS*-PV and *lukF*-PV genes, none of the ST398 isolates were positive for these genes. This is in accordance with studies in the United States that reported these genes in ST8 MRSA isolates [59, 63]. The presence of Panton-Valentine leucocidin producing genes is rare in LA-MRSA [24, 67]. Contrary to studies in Hong-Kong [25], none of the isolates carried the *egc* gene cluster (*seg*-*sei*-*sem*-*sen*-*seo*-*seu)* encoding staphylococcal enterotoxins. In addition, whereas all the ST8 isolates were positive for genes encoding enterotoxins K and Q, none of the ST398 isolates were positive for these enterotoxins. These genes are rarely detected in ST398 isolates [24, 67].

All the ST398 isolates in this study were resistant to tetracycline. Resistance to tetracycline is commonly associated with ST398 isolates [24, 68]. The tetracycline resistance phenotype observed in this study could be explained by the presence of the *tet*(K) and *tet*(M) genes. Tetracycline resistance is a hallmark of the livestock ST398 clades and is a well reported phenomenon in the literature [69]. In addition, multidrug resistance was observed in all isolates. A multi-resistance phenotype was reported in LA-MRSA isolates from retail poultry products in Germany [24] in accordance with this observation. Indeed, one of our ST398 isolate has resistance to tobramycin, which was found to be conferred by an *aad*(D) gene located on plasmids in agreement with previous findings [70]. This isolate was susceptible to all other aminoglycosides tested, which could be explained by the absence of the *aacA-aphD* gene in the microarray [71]. As previously reported, the *lnu*(A) gene encoding resistance to lincosamide was detected on plasmids in all ST398 isolates [72]. This resistance was shown to be conferred by the *spc* gene detected by PCR. Analysis of whole genome sequence data from an isolate (E154) with resistance to spectinomycin has indicated that the *spc* gene was linked to *erm*(A) (Usongo *et al*, unpublished). This was also observed with MRSA ST398 isolates from retail chicken products in Germany [24]. Despite the carriage of the *erm*(A) gene, all the ST398 isolates were susceptible to erythromycin, suggesting absence of a functional gene. The *fos*(B) gene encoding resistance to fosfomycin was detected only in ST8 isolates. This is in accordance with a Belgian study that reported this gene only in non-ST398 isolates [73]. Despite the presence of this gene, all the ST8 were susceptible to fosfomycin. Resistance to fosfomycin has mostly been reported in clinical settings owing to the high usage of this antimicrobial in human medicine [74]. Interestingly, the *qac*(C) gene encoding resistance to antiseptics and disinfectants was detected in the ST398 isolates.

To our knowledge, we report for the first time on biofilm formation in poultry MRSA isolates. Using slightly different experimental conditions, swine LA-MRSA isolates of ST398, ST9 and ST5 and human clinical isolates were also reported to form similar biofilms [75]. No correlation between *hld* gene expression, (*i*.*e*., a marker for Agr activation) and biofilm production was observed in our isolates, as previously reported in some strains of *S*. *aureus* from bovine mastitis [76]. Most of the probes targeting genes encoding MSCRAMMs, as well as the *icaADBC* operon, were positive with the microarray. Their presence suggests that these MRSA isolates have a good genetic capacity for adhesion to the extracellular matrix of the host and also for intercellular adhesion involved in biofilm formation. This likely favors colonization, persistence and zoonotic potential.

The identification of ST8, a human associated ST predominant in CA-MRSA [77] suggests likely contamination of retail chicken meat during slaughtering, processing or packaging as proposed previously [23, 28, 63]. However, because ST398 has previously been reported in broilers [11], and ST8 reported in live swine [8, 15, 78], these strains could also origin from the broilers. Interestingly, ST5 was recently reported from a poultry-adapted strain that originated from humans [79]. Whether this is the case for ST8 will require additional research.

## Conclusions

Our study shows the presence of MRSA in retail chicken meat. Two separate lineages of MRSA in retail chicken meat were observed, one of which is likely of human origin. The detected isolates possess genes encoding antibiotic resistance and virulence factors. In addition, the ability of these MRSA isolates to form biofilm could lead to colonization and persistence of these strains within the retail meat processing environment and this could enhance their zoonotic potential. While the role of retail meat as a vehicle of transmission of MRSA is still unknown and debatable, the fact that potential virulent strains were isolated from retail meat implies that this mode of transmission requires serious attention.

## Supporting information

S1 Dataset Dataset including the information on samples collection at the abattoir (“Sampling-abattoir”) and at the grocery stores (“Sampling-grocery”), and on results obtained for each isolate regarding biofilm formation (“Biofilms-DO”), *hld* expression (“Biofilm_Ct), DNA microarrays (“Micro-array”) and antimicrobial susceptibility testing (“AMR”). (XLSX)

S1 Fig Biofilm production in retail chicken meat MRSA isolates (*n =* 15) and reference strains. Results are given as *A*_490 nm_ mean of three independent biological replicates with SD. Red dot, strong biofilm production; green dot, moderate biofilm production; blue dot, weak biofilm production; gray dot, no biofilm production.
(TIF)

S2 Fig *hld* gene expression of representative retail chicken meat MRSA isolates. Strain 100N is used as a reference strain. Dashes represent the mean fold of three independent biological replicates for each isolate.
(TIF)

S1 Table Primer sequences used in this study. (DOCX)
