# Supplementary figures and images for: Prevalence and characteristics of Livestock-Associated Methicillin-Resistant Staphylococcus aureus (LA-MRSA) isolated from chicken meat in the province of Quebec, Canada
Source: PLoS One. 2020 Jan 10;15(1):e0227183. doi: 10.1371/journal.pone.0227183 (PMC6953868; doi:10.1371/journal.pone.0227183)

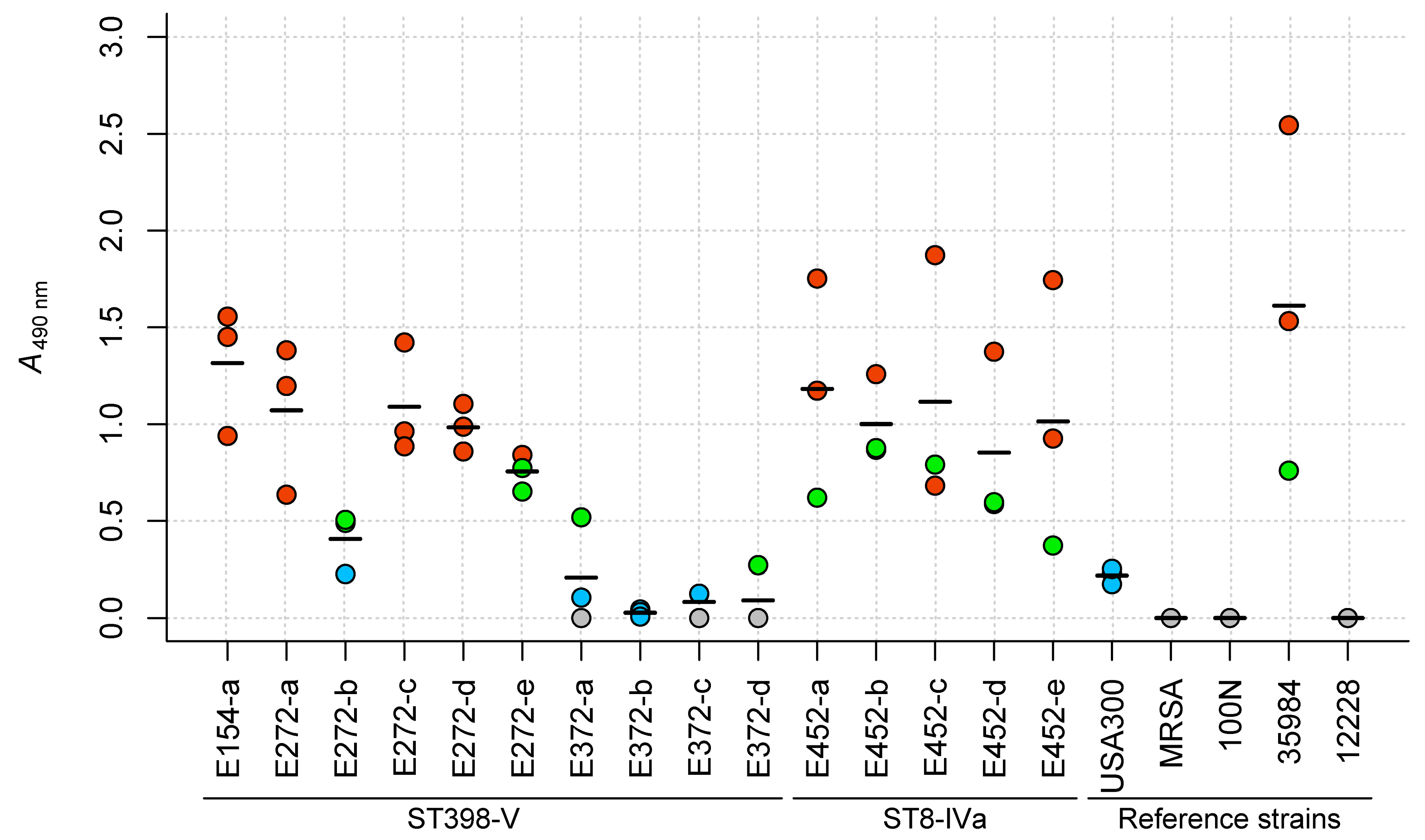

Supplement: S1 Fig — Results are given as A490 nm mean of three independent biological replicates with SD. Red dot, strong biofilm production; green dot, moderate biofilm production; blue dot, weak biofilm production; gray dot, no biofilm production. (TIF) [file pone.0227183.s002.tif]

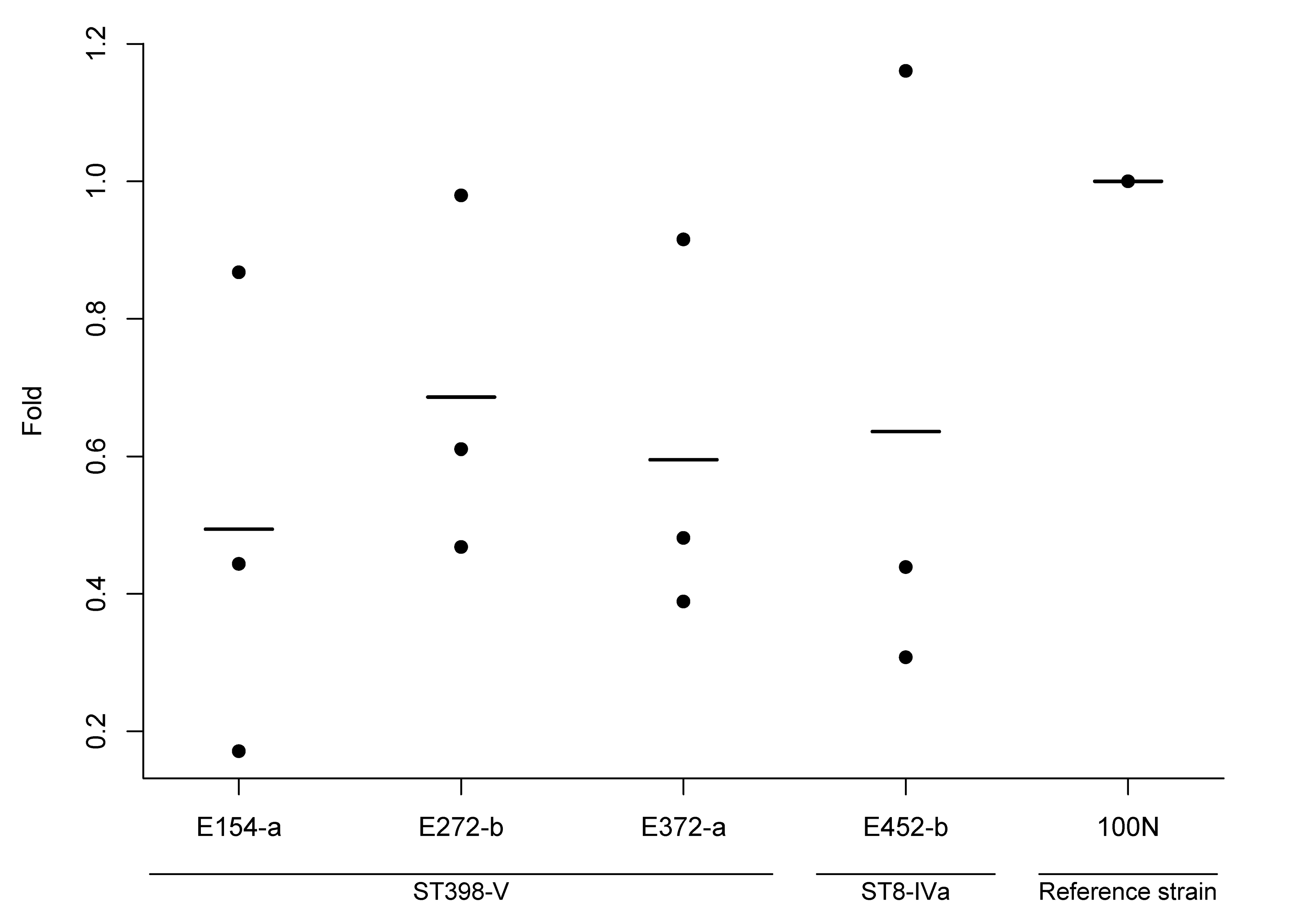

Supplement: S2 Fig — Strain 100N is used as a reference strain. Dashes represent the mean fold of three independent biological replicates for each isolate. (TIF) [file pone.0227183.s003.tif]
