# Supplementary material for: Prevalence and characteristics of Livestock-Associated Methicillin-Resistant Staphylococcus aureus (LA-MRSA) isolated from chicken meat in the province of Quebec, Canada
Source: PLoS One. 2020 Jan 10;15(1):e0227183. doi: 10.1371/journal.pone.0227183 (PMC6953868; doi:10.1371/journal.pone.0227183)
Supplement: S1 Table — (DOCX) [file pone.0227183.s004.docx]

**S1 Table. Primer sequences used in this study.**

| **Gene** | **Oligonucleotide primer (5’-3’)** | **PCR product (bp)** | **Reference** |
| --- | --- | --- | --- |
| **PCR** | | | |
| *mecA*-F | ACTGCTATCCACCCTCAAAC | 163 | [1] |
| *mecA*-R | CTGGTGAAGTTGTAATCTGG |  |  |
| *nuc*-F | GCGATTGATGGTGATACGGTT | 267 | [2] |
| *nuc*-R | AGC CAA GCC TTG ACG AAC TAA AGC |  |  |
| *mecC*-F | GCTCCTAATGCTAATGCA | 304 | [3] |
| *mecC*-R | TAAGCAATAATGACTACC |  |  |
| **qPCR** | | | |
| *gyrB*-F | GGTGCTGGGCAAATACAAGT | N/A | [4] |
| *gyrB*-R | TCCCACACTAAATGGTGCAA |  |  |
| *hld*-F | TAATTAAGGAAGGAGTGATTTCAATG | N/A | [4] |
| *hld*-R | TTTTTAGTGAATTTGTTCACTGTGTC |  |  |
| **Southern blot hybridization** | | | |
| *aadD*-F | GGAAGTGAATTTTGATAGCG | 438 | [5] |
| *aadD*-R | CTCAGAGTCGGAAAGTTGAC |  |  |
| *lnuA*-F | GGTGGCTGGGGGGTAGATGTATTAACTGG | 323 | [1] |
| *lnuA*-R | GCTTCTTTTGAAATACATGGTATTTTTCGATC |  |  |
| *tet(*K)-F | GATCAATTGTAGCTTTAGGTGAAGG | 155 | [6] |
| *tet(*K)-R | TTTTGTTGATTTACCAGGTACCATT |  |  |
| *tet*(M)-F | TGGTGGAATGATAGCCCATT | 406 | [6] |
| *tet*(M)-R | CAGGAATGACAGCACGCTAA |  |  |
| *spc*-F | AATGGTGGTTTACGCATTAACAGCG | 450 | [7] |
| *spc*-R | ATTCTGCAGCGACATCTTTCGAGG |  |  |

**References**

1. Lina G, Quaglia A, Reverdy ME, Leclercq R, Vandenesch F, Etienne J. Distribution of genes encoding resistance to macrolides, lincosamides, and streptogramins among staphylococci. Antimicrob Agents Chemother. 1999;43(5):1062-6. doi: 10.1016/S1684-1182(10)60081-3

2. Cho JI, Jung HJ, Kim YJ, Park SH, Ha SD, Kim KS. Detection of methicillin resistance in Staphylococcus aureus isolates using two-step triplex PCR and conventional methods. J Microbiol Biotechnol. 2007;17(4):673-6.

3. Cuny C, Layer F, Strommenger B, Witte W. Rare occurrence of methicillin-resistant *Staphylococcus aureus* CC130 with a novel mecA homologue in humans in Germany. PLoS One. 2011;6(9):e24360. doi: 10.1371/journal.pone.0024360

4. Allard M, Moisan H, Brouillette E, Gervais AL, Jacques M, Lacasse P, et al. Transcriptional modulation of some *Staphylococcus aureus* iron-regulated genes during growth in vitro and in a tissue cage model in vivo. Microbes Infect. 2006;8(7):1679-90. doi: 10.1016/j.micinf.2006.01.022

5. Trad S, Allignet J, Frangeul L, Davi M, Vergassola M, Couve E, et al. DNA macroarray for identification and typing of *Staphylococcus aureus* isolates. J Clin Microbiol. 2004;42(5):2054-64. doi: 10.1128/JCM.42.5.2054-2064.2004

6. Malhotra-Kumar S, Lammens C, Piessens J, Goossens H. Multiplex PCR for simultaneous detection of macrolide and tetracycline resistance determinants in streptococci. Antimicrob Agents Chemother. 2005;49(11):4798-800. doi: 10.1128/AAC.49.11.4798-4800.2005

7. Sartakova ML, Dobrikova EY, Terekhova DA, Devis R, Bugrysheva JV, Morozova OV, et al. Novel antibiotic-resistance markers in pGK12-derived vectors for *Borrelia burgdorferi*. Gene. 2003;303:131-7.
